# The sweet road to Parkinson’s disease

**DOI:** 10.18632/aging.101806

**Published:** 2019-02-01

**Authors:** Justine Renaud, Nicola Simola, Maria-Grazia Martinoli

**Affiliations:** 1 Cellular Neurobiology, Department of Medical Biology, Université du Québec, Trois-Rivières, Canada; 2 Department of Biomedical Sciences, Università di Cagliari, Cagliari, Italy; 3 National Institute of Neuroscience, Università di Cagliari, Cagliari, Italy; 4 Department of Psychiatry and Neuroscience, Université Laval and CHU Research Center, Québec, Canada

**Keywords:** hyperglycaemia, Parkinson’s disease, neurodegeneration, dopamine, diabetes, oxidative stress, aging

Long has functional neuroimagery exposed the importance of glucose in the fulfilment of the adult brain’s energetic needs. Actually, the brain utilizes 25% of total body glucose, mostly to power oxidative metabolism. However, the possible effects of hyperglycaemia on cerebral homeostasis are still contradictory. The clinical significance of hyperglycaemia in the brain emerged upon its formal identification as the prime culprit in comorbid diabetic complications [1]. The most affected collateral targets in diabetes mellitus are the kidneys, the cardiovascular system and the nervous system. Although it is clear that glucose concentrations rise in these cells under conditions of hyperglycaemia, how exactly cellular damage occurs is not as forthright. The current consensus is that oxidative stress explains hyperglycaemia-induced diabetic complications [2]. *Per se*, oxidative stress arises from an imbalance between the generation and clearance of reactive oxygen species (ROS), in favor of the former, and may ultimately provoke cell death. This inability to cope with an oxidative overload constitutes a key pathological element, not only in hyperglycaemia, but also in Parkinson’s disease (PD) and aging. Indeed, it is now well appreciated that oxidative stress is a key player in the nigrostriatal degeneration observed in PD [3], a neurological condition affecting an estimated 1% of the population over the age of 60 years, making it the second most common neurodegenerative disorder after Alzheimer’s disease. PD pathology features a progressive loss of dopaminergic neurons harbored in the substantia nigra pars compacta (SNc) of the midbrain, which leads to decreased dopamine release in the dorsal striatum responsible for the emergence of motor impairments. The first accounts of a possible association between diabetes and PD date back to almost 60 years ago. Since then, other studies showed that diabetes exacerbates the progression of motor and cognitive deficits in PD and that drug-naïve parkinsonian patients display higher-than-normal levels of fasting blood glucose within the pre-diabetic diagnostic range [4].

Despite arduous efforts deployed to improve our understanding of the neuropathological underpinnings of PD, researchers are still at loss as to why the nigrostriatal dopaminergic pathway undergoes preferential early degeneration compared, for instance, to the neighboring mesocorticolimbic pathway originating from the ventral tegmental area (VTA) in the midbrain and projecting to the ventral striatum and prefrontal cortex. Rapidly gaining momentum is a proposition providing that nigrostriatal dopaminergic neurons possess a distinctive phenotypic liability responsible for their relative susceptibility to oxidative stress. Specifically, the SNc holds high levels of iron ions that promotes the generation of highly reactive hydroxyl radicals but also possesses low levels of the ubiquitous antioxidant molecule glutathione. Moreover, dopaminergic nigrostriatal neurons are endowed with an exceptionally dense dendritic arborization, rich in mitochondria and constantly requiring costly metabolic sustenance. On these bases, we hypothesized that nigrostriatal dopaminergic neurons are more vulnerable to hyperglycaemia-induced oxidative stress compared to other neuronal populations, expressly ones of the mesocorticolimbic pathway. We began by verifying that high glucose settings induce the death of dopaminergic neurons in culture and that this degeneration is linked to oxidative stress. Indeed, dopaminergic neurons in culture promptly exhibited high levels of superoxide anion, a key ROS whose overproduction constitutes the earliest event in hyperglycaemia-induced oxidative stress, as well as downregulated superoxide dismutase activity [5,6]. Continued high glucose conditions led to the apoptotic death of dopaminergic cells validated by DNA fragmentation and altered expression profiles of various markers of apoptosis such as nuclear translocation of apoptosis inducing factor (AIF), Poly [ADP-ribose] polymerase (PARP) and caspase-3 cleavage [5,6]. We then set out to verify our central hypothesis in a rat model of long-term hyperglycaemia. We employed a paradigm that utilizes streptozotocin, a toxin that targets insulin-producing pancreatic β cells, to generate a model presenting a hyperglycaemic phenotype that could be maintained for up to 6 months. Employing immunohistochemical and immunoblotting techniques, we demonstrated that long-term hyperglycaemia in rats causes the degeneration of dopaminergic neurons in the SNc, but not in the VTA [7]. Accordingly, dopaminergic terminal fibers were less dense in the dorsal than in the ventral striatum of hyperglycaemic rats. By intracerebral microdialysis technique, we also showed that dopamine release was diminished in the dorsal striatum, but not in the ventral striatum or in the prefrontal cortex. We further discovered a noticeable increase in astrocytes that was neuroanatomically coincidental to a loss of microglial cells in the SNc and striatum, but not in the VTA. Behavioural alterations were assessed in a series of tasks designed to uncover motor deficits in rodent models of PD. Long-term hyperglycaemic rats manifested signs of bradykinesia and gait disturbances reminiscent of parkinsonian motor impairments [7]. Interestingly, motor deficits and dampened dorsostriatal dopamine release were apparent before neurodegeneration could be discerned, suggesting possible functional impairments of the nigrostriatal pathway before of neuronal death.

Our data clearly reveal that hyperglycaemia is sufficient to induce nigrostriatal dopaminergic degeneration and expose the preferential vulnerability of the nigrostriatal pathway to sustained hyperglycaemia, supporting the physiological significance of their phenotypic liability to oxidative stress. Our findings further strengthen the apparent epidemiological link between pre-existing diabetes and an increased risk of developing PD and support the clinical efforts to tighten glycaemic control in PD patients.
